# Effects of Jaw Clenching While Wearing a Bite‐Aligning Mouthguard on Performance in Athletes: A Systematic Review

**DOI:** 10.1155/ijod/2919955

**Published:** 2026-07-02

**Authors:** Alice Cichelli, Rosita Delfina Valloreo, Maurizio Bertollo, Domenico Tripodi, Mirko Pesce, Simonetta D’Ercole, Teresa Paolucci

**Affiliations:** ^1^ Department of Medical, Oral and Biotechnological Sciences, “G. d’Annunzio” University of Chieti-Pescara, Chieti, 661100, Italy, unich.it; ^2^ Department of Engineering and Geology, “G. d’Annunzio” University of Chieti-Pescara, Pescara, 65127, Italy, unich.it; ^3^ Department of Medicine and Aging Sciences, “G. d’Annunzio” University of Chieti-Pescara, Chieti, 66100, Italy, unich.it; ^4^ BIND (Behavioural Imaging and Neural Dynamics Center), “G. d’Annunzio” University of Chieti-Pescara, Chieti, 66100, Italy, unich.it; ^5^ C.A.R.E.S. – Center for Disability, Rehabilitation and Sports Medicine, “G. d’Annunzio” University of Chieti-Pescara, Chieti, 66100, Italy, unich.it

**Keywords:** aerobic capacity, athletic performance, exercise, mouthguards, strength

## Abstract

**Background:**

Emerging evidence suggests functional connectivity between the masticatory and musculoskeletal systems, yet the extent to which jaw clenching modulates athletic performance remains uncertain. Bite‐aligning mouthguards (MGs) are designed to optimize occlusion and stability, but systematic quantification of their impact on performance across athletes is limited. It is important to distinguish between conventional protective MGs and performance‐oriented devices designed to modify mandibular position, as their mechanisms and potential effects may differ. This review synthesizes available data to clarify whether jaw clenching with bite‐aligning devices enhances strength, power, endurance, precision, or neuromuscular efficiency.

**Objectives:**

The objective of this study was to systematically review the literature on the effect of bite‐aligning MGs on athletic performance.

**Materials and Methods:**

A comprehensive search of PubMed, Scopus, and Web of Science was conducted until 1 February 2024. Inclusion criteria were (1) randomized controlled trials (RCTs), (2) crossover trials, and (3) controlled clinical trials evaluating MGs’ effects on muscle strength, power, agility, quickness, and sports performance. A narrative synthesis approach was adopted due to heterogeneity in study design, interventions, and outcome measures, which precluded meta‐analysis.

**Results:**

Thirteen trials with 335 athletes (mean age: 24.13 ± 5.64 years) were included. At least four studies reported positive effects on some variables, while none reported negative effects. Main findings indicate neuromuscular benefits, particularly in rugby, football, and weightlifting. Custom‐made MGs (CMGs) showed a particularly positive impact. Neutral results were also observed, showing no difference between using a MG or not.

**Conclusions:**

Athletes may benefit from wearing MGs not only to prevent orofacial trauma but also to potentially enhance performance in specific actions. Benefits are associated with increased peak acceleration, force, muscle activity, and power in upper‐ and lower‐body exercises. However, given the high heterogeneity across studies, these findings should be interpreted with caution, and the effects cannot be considered universal. Further high‐quality studies are required to clarify the potential performance‐enhancing role of specific MG designs.

## 1. Introduction

A mouthguard (MG) is a dental device that covers the teeth and surrounding tissue, primarily to prevent or reduce trauma to the teeth, gums, lips, and jaw. It is usually worn on the upper jaw and works by separating the upper and lower teeth, protecting them from the surrounding soft tissues. It also absorbs or redistributes shock and/or stabilizes the jaw during traumatic jaw closure. MGs are a key factor in preventing dental injuries related to sports, especially contact sports [1–3]. Several studies have convincingly demonstrated their preventive effect [4–7]. However, many athletes are reluctant to wear MGs, largely due to breathing restrictions [8, 9] and the fear of impaired performance [10]. Nevertheless, MG adherence is a multifactorial behavior influenced not only by physiological constraints but also by psychosocial determinants, including peer influence, team culture, social perception, and individual attitudes, particularly among young athletes [1]. These limitations appear to vary depending on the model. There are two main types of MG: custom‐made and inexpensive self‐adapted MGs (SAMGs). Custom‐made MGs (CMGs) are widely used across both amateur and professional sport settings and are individually fabricated by dental professionals, whereas SAMGs are designed for mass production and widespread use, especially in youth sports [11, 12]. It is also important to distinguish between conventional protective MGs and performance‐oriented devices designed to modify mandibular position (e.g., neuromuscular or bite‐aligning devices), as their functional mechanisms and potential effects on performance may differ [13, 14].

In the late 1970s and early 1980s, research suggested that MGs could provide protection against dental injuries and enhance performance. Smith et al. reported a correlation between muscular strength and proper temporomandibular joint (TMJ) alignment. Specifically, they observed that performance during isometric deltoid press testing significantly improved in a National Football League team of around 25 players when the mouth was positioned in a “wax bite” (similar to a boil‐and‐bite) compared to clenching the teeth [15]. Subsequent preliminary investigations conducted on small samples further explored this hypothesis, reporting improvements in muscular strength and neuromuscular coordination associated with mandibular repositioning strategies [16]. These improvements have not only been reported in terms of muscular strength but also in enhanced neuromuscular coordination and athletic performance [16]. Some reports have suggested that endurance athletes experience improvements when wearing a mouthpiece compared to not wearing one. These athletes provided subjective feedback, indicating an increased ability to train harder and faster recovery from injuries. However, due to the lack of reliable, controlled studies, these findings may be influenced by the placebo (PLA) effect. Conversely, more recent evidence has reported no significant impairment in aerobic performance associated with MG use, thereby providing a more balanced interpretation of the literature [17].

A study assessing airway openings during endurance exercise with and without a mouthpiece found that lactate levels decreased when subjects wore a mouthpiece compared to when they did not. In addition, subjects displayed increased upper airway cavity diameters with a mouthpiece vs. without [18]. Another study found that, when running at 75%–85% of maximum heart rate (HR), subjects (*N* = 24) who wore a mouthpiece had significantly lower lactate levels (4.01 and 4.92 mmol ^∗^L^−1^, respectively, *p* = 0.024, a 23% improvement) than those who did not wear a mouthpiece [19]. Where reported, lactate measurements refer to blood lactate concentrations, ensuring consistency in physiological interpretation across studies. The hypothesis is that there is some improvement in oxygen consumption, which appears to affect lactate levels during moderate‐intensity endurance exercise. Tahara et al. [20] found that clenching and biting could decrease cortisol release in humans during psychological stress. They discovered that subjects displayed a decreased cortisol response during a 20‐min mental stress activity when they were able to clench their teeth or chew on paraffin wax. Both of these actions (clenching and chewing) would require the use of the masticatory muscles. The authors suggest that activity in these muscles triggers a series of events that first activates the motor area of the cerebrum. This leads to a decreased hypothalamic–pituitary response, resulting in reduced cortisol release. In the studies included in this review, cortisol was primarily assessed using salivary samples unless otherwise specified, which is relevant for reproducibility and interpretation. However, there is little data to support or refute this response during a physiological stressor, such as high‐intensity exercise. Numerous researchers have demonstrated a more robust cortisol response to higher‐intensity resistance exercise [21–23]. McGuigan et al. found a 145% difference in salivary cortisol levels between low‐ (three sets) and high‐ (12 sets) intensity resistance training. Cortisol levels were found to be 97% higher after high‐intensity exercise [22].

Some studies have postulated that using a CMG has no negative effect on breathing (𝑉˙_E_), oxygen uptake (𝑉˙O_2_), or maximum performance compared to wearing a conventional SAMG or no MG at all [10, 13, 14, 24]. In activities involving high forces or requiring metabolic efficiency, even the use of a CMG has been shown to maximize ergogenic effects [18, 25, 26]. The hypothetical effects of CMG caused by an increase in airway diameter are described [18], showing positive effects on gas exchange [19, 27, 28] and enhancement through jaw repositioning, which is associated with beneficial effects on peripheral muscle innervation [13, 14, 25]. Hemodynamic parameters associated with MG use have not yet been measured. However, documenting these cardiac parameters could provide deeper insights into the effects of SAMG use, which may be closely associated with an increase in airway resistance [8, 9].

It is widely accepted that MGs are effective in protecting against injuries in sports. However, the effects of MGs on strength and power production remain controversial. Based on this, the objective of this systematic review was to investigate the effect of wearing MGs on athletic performance, considering the different protocols adopted and the type of bite used.

## 2. Materials and Methods

### 2.1. Protocol and Registration

This systematic review was conducted in accordance with the PRISMA Statements [29] and was prospectively registered in the PROSPERO database (registration ID: CRD42024506308).

### 2.2. Eligibility Criteria

The eligibility criteria were established using the PICOS model:

• Population (P): elite or professional athletes;
• Intervention (I): use of a protective MG during training or competition;
• Comparison (C): no use of MG or PLA device;
• Outcomes (O): athletic performance outcomes (breathing, strength, speed, and endurance);
• Study design (S): randomized controlled trials (RCTs), crossover trials, and controlled clinical trials.

The null hypothesis of this review was that the use of MGs does not significantly influence athletic performance compared to no‐MG conditions.

The exclusion criteria were editorials, letters, book chapters, pilot studies, theses, clinical guidelines, laboratory and animal studies, descriptive designs, such as case reports or case series, and articles not written in English. Inclusion and exclusion criteria were applied independently by two reviewers (AC and RDV), and any disagreements were resolved by consensus with a third reviewer (TP). Studies were grouped by outcome domain for qualitative synthesis (e.g., respiratory effects, strength, and speed).

### 2.3. Information Sources and Search Strategy

An extensive electronic search was performed on PubMed, Scopus, and Web of Science from the inception of the databases to 1 February 2024. The search strategy employed MeSH terms (“Mouth Protectors”) and related free‐text keywords selected from the MeSH and DeCS thesauri. Boolean operators (AND/OR) were used to combine the search terms (see Table 1 for the full strategy). A manual search of the reference lists of the included articles was also conducted.

**Table 1:** Electronic database search strategies.

Database	Search strategy
PubMed	(“Mouth Protectors” [MeSH] OR “mouthguard ^∗^” OR “mouth guard ^∗^” OR “oral protector ^∗^” OR “sports mouthpiece ^∗^”) AND (“athletic performance” OR strength OR endurance OR speed)
Scopus	TITLE‐ABS‐KEY (“mouthguard ^∗^” OR “mouth guard ^∗^” OR “oral protector ^∗^” OR “sports mouthpiece ^∗^”) AND (“athletic performance” OR strength OR endurance OR speed)
Web of Science	TS = (“mouthguard ^∗^” OR “mouth guard ^∗^” OR “oral protector ^∗^” OR “sports mouthpiece ^∗^”) AND TS = (“athletic performance” OR strength OR endurance OR speed)

*Note:* Search strategies were adapted to each database’s syntax. Searches were limited to English‐language articles. No filters regarding publication date or study design were applied. The search was supplemented by manual screening of the reference lists of included articles.

Due to the substantial heterogeneity in study design, intervention types (different MGs), and outcome measures, a meta‐analysis was not considered appropriate, and a narrative synthesis approach was therefore adopted.

### 2.4. Study Selection

After removing duplicates, two reviewers (AC and RDV) screened the titles and abstracts of the remaining articles for relevance. The full texts of potentially eligible articles were then assessed. Any disagreements were resolved through consensus with a third reviewer (TP). No automation tools were used in the selection process.

### 2.5. Data Extraction

Two reviewers extracted the data independently using a standardized Microsoft Excel spreadsheet. Any discrepancies were resolved by consensus, with the involvement of a third reviewer when necessary. The following data were collected:

• First author and year of publication;
• Country of origin;
• Sample size, age, and gender of participants;
• Type of sport;
• Type of MG used;
• Comparator;
• Outcome measures;
• Main findings related to athletic performance.

Attempts were made to contact the study authors when relevant data were missing or unclear.

### 2.6. Risk of Bias Assessment and Methodological Quality

Two reviewers independently evaluated the methodological quality of the RCTs using the Physiotherapy Evidence Database (PEDro) scale [30]. Studies were categorized as follows: excellent (9–10 points), good (6–8 points), fair (4–5 points), or poor (<4 points). Any disagreements were resolved by reaching a consensus with a third reviewer. Additionally, the Cochrane risk of bias tool was used to assess the following eight domains: (1) random sequence generation, (2) allocation concealment, (3) blinding of participants and personnel, (4) blinding of outcome assessors, (5) incomplete outcome data, (6) selective reporting, (7) description of inclusion/exclusion criteria, and (8) presence of a priori sample size calculation. Each domain was rated as low risk (+), high risk (−), or unclear (?). Studies with two or more domains rated as high risk were considered to have an overall high risk of bias [31].

### 2.7. Effect Measures

For continuous outcomes (e.g., strength or speed), mean or standardized mean differences were considered. Where reported, confidence intervals and standard deviations were extracted and reported in the specific tables.

### 2.8. Data Synthesis

A narrative synthesis was carried out due to heterogeneity in intervention protocols, MG types, and outcomes assessed. This heterogeneity precluded the possibility of performing a meta‐analysis, and therefore, results were organized thematically according to the outcome measured. No subgroup analysis or sensitivity analysis was performed.

## 3. Results

**Figure 1:**
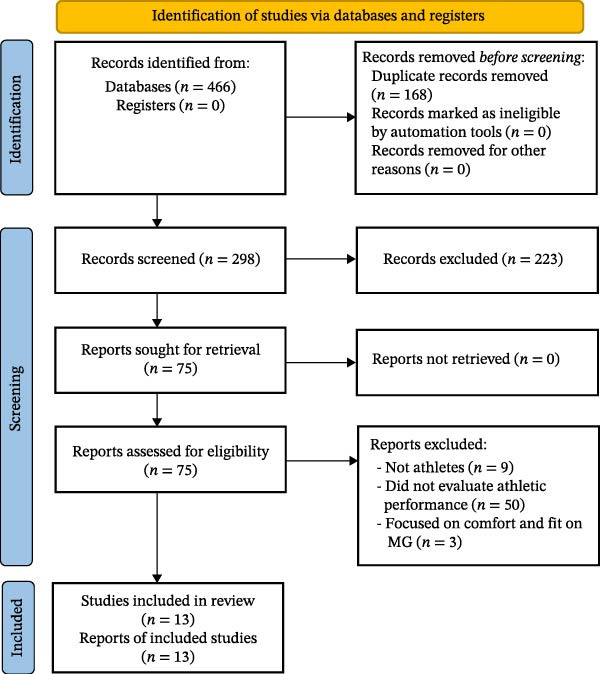
PRISMA 2020 flow diagram for the systematic review [32].

The electronic database search yielded 466 articles. After removing 168 duplicates, 298 records were screened based on their titles and abstracts, and 223 articles were excluded. This process resulted in 75 articles being selected for full‐text review as potentially eligible. During the full‐text assessment, 62 articles were excluded as they did not meet the inclusion criteria. Consequently, 13 studies were included in the systematic review (see Figure 1) [27, 33–44].

The included studies comprised both RCTs and controlled clinical trials and were published between 2006 (Bourdin et al.) [35] and 2022 (Dias et al.) [38]. Two of the studies were conducted in Brazil [33, 40], seven in the United States [27, 33, 36, 37, 39, 42, 43], one in France [35], one in Portugal [38], one in Germany [41], and one in Australia [44].

### 3.1. Characteristics of Included Studies

The 13 included controlled studies (including RCTs and controlled clinical trials) involved a total of 335 athletes. The sample sizes in the individual studies ranged from 10 [36] to 50 [43] athletes in both the experimental and control groups. The mean age of participants ranged from 17 years [40] to 32 years [39], with an overall mean age of 24.13 ± 5.64 years.

The athletes were involved in a wide range of sports, including karate, rugby, football, rowing, futsal, handball, soccer, basketball, track and field, volleyball, lacrosse, hockey, water polo, and weightlifting. This variability in sports disciplines reflects differences in physical demands and performance outcomes, contributing to the heterogeneity observed across the included studies. The main characteristics of the included studies are summarized in detail in Table 2.

**Table 2:** Summary of the characteristics of the included studies.

Author (year)	Study type	Athletes	Number/gender	Mean age	Sport
Raquel et al. (2015) [33]	Clinical trial	20	14 M 6 F	23.7 ± 7.5 y.o.	Karate
Cotter et al. (2017) [34]	Clinical trial	42	34 M 8 F	21.9 ± 2.9 y.o.	Mixed sports disciplines (*n* = 20 categories)
Bourdin et al. (2006) [35]	Randomized controlled trial	19	19 M	27 ± 4.8 y.o.	Team sports
Drum et al. (2016) [36]	Randomized controlled trial	10	10 M	20.7 ± 0.8 y.o.	Football
Duddy et al. (2012) [37]	Randomized controlled trial	18	18 M	NR (range: 19–23 y.o.)	Rowing
Dias et al. (2022) [38]	Randomized controlled trial	22	22 M	25 ± 3.8 y.o.	Rugby
Gage et al. (2015) [39]	Randomized controlled trial	24	14 M 10 F	32.2 ± 7.3 y.o.	Weightlifting
Collares et al. (2014) [40]	Randomized controlled trial	40	40 M	NR (range: 15–17 y.o.)	Futsal and soccer
Lässing et al. (2021) [41]	Randomized controlled trial	15	NR	17 ± 0.5 y.o.	Handball
Garner et al. (2011) [27]	Randomized controlled trial	28	28 M	NR (range: 18–22 y.o.)	Football
Golem et al. (2017) [42]	Randomized controlled trial	20	20 M	NR (range: 18–21 y.o.)	NR
Dunn‐Lewis et al. (2012) [43]	Randomized controlled trial	50	26 M 24 F	24.0 ± 3.7 y.o.	Basketball, soccer, track and field, volleyball, lacrosse, football (men), rugby
Gebauer (2011) [44]	Randomized controlled trial	27	27 M	23.5 ± 3.8 y.o.	Hockey or water polo

*Note:* Reported data include author and year of publication, study type, total number of athletes, gender distribution, mean age ± standard deviation (where available), and sport practiced.

Abbreviations: F, female; M, male; NR, not reported; y.o., years old.

### 3.2. Effects of MGs on Athletic Performance (Outcomes)

Analysis of the 13 controlled studies (including RCTs and controlled clinical trials) revealed a wide range of outcomes related to the use of MGs. The studies primarily evaluated the impact of MGs on parameters such as strength, endurance, and neuromuscular performance.

Overall, the findings were heterogeneous and did not consistently demonstrate a clear performance–enhancing effect of MG use. While some studies reported improvements in specific variables, others showed no significant differences compared to control conditions.

In particular, CMGs and performance‐oriented devices appeared to be associated with more favorable outcomes in certain contexts; however, these effects were not uniform across all studies and should be interpreted with caution due to variability in study design, participant characteristics, and outcome measures.

The detailed outcomes of each study are presented in Table 3.

**Table 3:** Effects of MGs on sporting performance‐related outcomes.

Author (year)	Mouthguard	Test	Outcomes
Raquel et al. (2015) [ 33]	1) No MG 2) RMG 3) CMG	1) sEMG of temporal and masseter muscles at rest, clenching, MVC before/after training 2) Same 3) Same	1) Baseline EMG activity 2) No significant EMG changes 3) ↓ EMG in temporal muscles after training; preserves EMG profile pre/posttraining
Cotter et al. (2017) [34]	BB NMDD	FMS (functional movement screen) mSEBT (modified star excursion balance test) SLL (single‐leg landing)	NMDD ineffective in altering dynamic movement compared to BB or NO conditions
Bourdin et al. (2006) [35]	SAMG CMG	Visual reaction time, explosive power, ventilation, and oxygen consumption	No effect of MG on main physiological parameters
Drum et al. (2016) [36]	CMG BB No MG	Blood lactate concentration, peak fatigue, flexibility, squat vertical jump, maximum bench press	No superior effect of CMG on general fitness vs. BB and No MG
Duddy et al. (2012) [37]	BB CMG	Ergometer test	CMG had no detrimental effect on athletic strength and performance
Dias et al. (2022) [ 38]	No MG MCM NCM OS	Bench press test	MCM: significantly higher peak acceleration and peak force
Gage et al. (2015) [39]	SAMG CMG No MG	Maximum power clean lift	Perceived greater strength with CMG
Collares et al. (2014) [40]	CMG	Shuttle‐run test	No influence of CMG on aerobic performance
Lässing et al. (2021) [41]	nCMG CMGvent No MG	Spirometry, blood lactate, cortisol	No differences across conditions
Garner et al. (2011) [27]	CMG No MG	Intense resistance exercise	­↑ Cortisol levels in CMG vs No MG
Golem et al. (2017) [42]	No MG PLA SAMG CMG	Rest: respiratory flow dynamics, MVV Exercise: graded maximal treadmill test, peak lactate	Rest: no MG ↑­ peak expiratory flow vs. others; PLA and SAMG ↓ MVV vs. no MG Exercise: no differences in ventilation, VO_2,_ VCO_2_, peak lactate
Dunn‐Lewis et al. (2012) [43]	1) PBMG: customized OTC Power Balance performance MG 2) Reg MG: regular OTC boil‐and‐bite MG 3) No MG: control	Sit‐and‐reach flexibility, medial‐lateral balance, visual reaction time, vertical jump, 10‐m sprint, bench throw, plyo press power quotient, HR, and RPE recorded around 3PQ	Bench throw power and force ­↑ with PBMG vs. Reg MG and no MG in men and women. In men only: 3PQ power and force ↑­ with PBMG vs. others; vertical jump rate of power development ↑­ with PBMG. No differences in flexibility, balance, reaction time, sprint, HR, or RPE
Gebauer et al. (2011) [ 44]	1) Custom laminated MG with normal palatal surface 2) Custom laminated MG with palatal coverage up to gingival margin 3) No MG	Graded exercise test on treadmill; ventilation, oxygen uptake at 10 km ^∗^h^-1^, 12 km ^∗^h^-1^, and at VO_2_ peak	No significant differences in ventilation or VO_2_ between conditions at any exercise intensity

Abbreviations: 3PQ, plyo press power quotient; BB, boil‐and‐bite mouthguard; CMG, custom‐made mouthguard; CMGvent, custom‐made mouthguard with respiratory channels; HR, heart rate; MCM, controlled mouthguard; MVC, maximum voluntary contraction; MVV, maximum voluntary ventilation; NCM, noncontrolled mouthguard; nCMG, custom‐made mouthguard without respiratory channels; NMDD, neuromuscular dentistry–designed mouthguard; OS, occlusal splint; OTC, over the counter; PLA, placebo; RMG, ready‐made mouthguard; RPE, rating of perceived exertion; SAMG, self‐adapted mouthguard; sEMG, surface electromyography.

### 3.3. Study Quality

We assessed the study quality according to the PEDro scale [30], reporting that eight studies (62%) [27, 34, 36–38, 42–44] were of good methodological quality, and five studies (38%) [33, 35, 39–41] were of fair methodological quality (quality scoring for each assessment criterion is shown in Table 4). These classifications refer to the included controlled studies (including RCTs and controlled clinical trials), rather than exclusively randomized designs.

**Table 4:** Methodological quality assessment of included studies (PEDro scale).

Study (author, year)	PEDro score (0–10)	Quality category
Raquel et al. (2015) [33]	4	Fair
Cotter et al. (2017) [34]	8	Good
Bourdin et al. (2006) [35]	5	Fair
Drum et al. (2016) [36]	8	Good
Duddy et al. (2012) [37]	6	Good
Dias et al. (2022) [38]	7	Good
Gage et al. (2015) [39]	4	Fair
Collares et al. (2014) [40]	5	Fair
Lässing et al. (2021) [41]	5	Fair
Garner et al. (2011) [27]	7	Good
Golem et al. (2017) [42]	7	Good
Dunn‐Lewis et al. (2012) [43]	6	Good
Gebauer et al. (2011) [44]	6	Good

The risk of bias among the included studies was estimated using the Cochrane risk of bias tool (see Table 5). Regarding random sequence generation, all 13 studies (100%) were judged to have an adequate or reported randomization process. Blinding of participants and personnel (performance bias) was adequately addressed in only two studies (15%) [34, 36], while complete outcome data (attrition bias) were reported in six studies (46%) [34, 36, 38, 39, 42, 43]. Only two studies (15%) [34, 36] clearly described blinding of outcome assessment (detection bias), and nine studies (69%) [33, 34, 36–43] were considered at low risk of selective reporting bias.

**Table 5:** Risk of bias of the included studies.

Study	Risk of bias domains								
	D1	D2	D3	D4	D5	D6	D7	D8	Overall
Raquel et al. [33]	−	−	−	−	?	+	+	−	−
Cotter et al. [34]	+	+	+	+	?	+	+	?	+
Bourdin et al. [35]	+	?	−	−	−	+	+	−	−
Drum et al. [36]	+	?	+	+	+	+	+	−	+
Duddy et al. [37]	+	+	−	−	?	+	+	+	−
Dias et al. [38]	+	?	−	?	+	+	+	+	+
Gage et al. [39]	+	−	−	−	+	+	+	−	−
Collares et al. [40]	+	−	−	−	−	+	+	+	−
Lässing et al. [41]	+	−	−	−	−	+	+	+	−
Garner et al. [27]	+	+	−	?	+	+	+	?	+
Golem et al. [42]	+	?	−	?	+	+	+	?	+
Dunn‐Lewis et al. [43]	+	?	−	−	+	+	+	?	−
Gebauer et al. [44]	+	?	−	−	?	+	+	+	−

*Note*: Domains: (D1) random sequence generation; (D2) allocation concealment; (D3) blinding of participants and personnel; (D4) blinding of outcome assessors; (D5) incomplete outcome data; (D6) selective reporting; (D7) description of inclusion/exclusion criteria; and (D8) presence of a priori sample size calculation. Each domain was rated as low risk (+), high risk (−), or unclear (?).

Overall, although the majority of studies demonstrated acceptable methodological quality, several domains of bias were either inadequately reported or unclear, which should be taken into account when interpreting the findings.

## 4. Discussion

This systematic review aimed to investigate the effect of wearing MGs on athletic performance when different protocols and bite types were considered. The findings from the included studies provide a mixed, but predominantly neutral, view of the relationship between MG use and athletic performance. The majority of studies concluded that wearing a MG does not have a negative impact on key physiological parameters or athletic performance. Several studies, including those by Cotter et al. [34], Bourdin et al. [35], and Drum et al. [36], have demonstrated that wearing a MG (whether custom‐made or boil‐and‐bite) does not affect the main physiological parameters associated with team sports performance compared to not wearing a MG. Similar findings were reported by Collares et al. [40], who found that custom‐fit MGs did not affect the aerobic performance of football and futsal players. Lässing et al. [41] also showed that, under stress, there were no differences in cortisol, ventilation, cardiac, and metabolic responses when using a CMG (with or without respiratory channels) compared to not wearing a MG.

However, a minority of studies suggest that certain types of MG, particularly those with a specific design or occlusal configuration, may improve performance. For instance, Dias et al. [38] discovered that athletes using a controlled MG (MCM) exhibited significantly higher peak acceleration and force during a ballistic bench press than those not using a MG. Similarly, Dunn‐Lewis et al. [43] reported that a “Power Balance” performance MG improved the performance of both men and women in upper‐body loaded power exercises and men only in lower‐body power exercises. Gage et al. [39] also found that the Power Balance MG produced greater muscle activity than another self‐fit performance MG. These findings are supported by Garner et al. [27], who observed a significant decrease in cortisol levels 10 min after exercise in subjects wearing a MG compared to those not wearing a MG.

Importantly, these heterogeneous findings may be partially explained by the fundamental differences between conventional protective MGs and performance‐oriented devices designed to alter the mandibular position. While standard MGs primarily serve a protective function, “bite‐aligning” or neuromuscular devices aim to optimize occlusion and TMJ positioning, potentially influencing neuromuscular output. This distinction is critical for interpretation, as pooling these devices under a single category may obscure device‐specific effects.

From a mechanistic perspective, the so‐called “jaw repositioning” hypothesis has been proposed to explain the potential ergogenic effects of certain MGs. This hypothesis suggests that mandibular alignment may influence neuromuscular coordination through modulation of trigeminal afferent input, which plays a key role in sensorimotor integration. Improved afferent feedback may enhance motor unit recruitment, postural stability, and force transmission. The relationship between sensorimotor integration, postural control, and musculoskeletal function has also been highlighted in rehabilitation research, suggesting that alterations in cranio‐cervical and mandibular positioning may influence global neuromuscular performance [45]. Experimental evidence supporting this mechanism has been reported by Haughey and Fine [46], who demonstrated that alterations in lower jaw position can influence strength and performance parameters in elite athletes. Although these findings are promising, the underlying neurophysiological pathways remain incompletely understood and require further investigation through controlled experimental studies.

Despite these potential mechanisms, the overall evidence remains inconsistent, and the high heterogeneity across studies must be carefully considered. Differences in study design, participant characteristics, types of MGs, and outcome measures limit the comparability of results and preclude definitive conclusions. Therefore, any interpretation suggesting performance enhancement should be considered with caution, and the results should not be generalized across all athlete populations or device types.

In this context, the findings of the present review do not allow a clear rejection of the null hypothesis, namely, that the use of MGs does not significantly influence athletic performance. While some studies report improvements in specific parameters, these effects are not consistent across all outcomes and are often dependent on device design and testing conditions.

These varying results suggest that the design and function of the MG, such as a “controlled bite” or “jaw repositioning” mechanism, may be crucial in enhancing the performance, setting them apart from standard custom‐made or boil‐and‐bite options.

### 4.1. Limitations of the Review

The evidence base for this review has several limitations. The included studies vary greatly in terms of their design, participant populations, and performance outcomes measured. The studies involve athletes from various sports, including handball, karate, football, and rugby, which have different physical demands. The performance parameters assessed also varied widely, ranging from electromyographic activity and aerobic performance to strength, power, and dynamic movement ability. Furthermore, the small sample sizes of many of the included studies may limit the generalizability and statistical power of their findings. The different types of MG tested (e.g., custom‐made, boil‐and‐bite, neuromuscular dentistry–designed mouthguard (NMDD), and performance‐specific models) also make it difficult to make direct comparisons or draw unified conclusions across all studies.

Additionally, the inability to perform a meta‐analysis represents an important limitation of this review. This was primarily due to the substantial heterogeneity in study protocols, intervention types, and outcome measures, which prevented meaningful quantitative synthesis. As a result, a narrative synthesis approach was adopted, which, although appropriate, may introduce a degree of subjectivity in the interpretation of the findings.

### 4.2. Implications for Future Research

The results of this review have clear implications. In practice, the consistent finding that MGs do not negatively affect performance should reassure athletes, coaches, and practitioners. The main purpose of a MG is to prevent injury, and this review suggests that this protective function does not negatively impact athletic performance.

Future research should specifically differentiate between protective and performance‐oriented MGs, adopting standardized protocols and clearly defined outcome measures. Greater emphasis should also be placed on the investigation of neuromuscular mechanisms underlying jaw repositioning and its potential impact on performance. Furthermore, studies should clearly define the athletic population under investigation, as the term “elite athletes” is often inconsistently applied across studies. In the present review, elite athletes should be considered as individuals competing at national or international level or engaged in structured high‐performance training programs.

In terms of policy, these findings support the continued recommendation of wearing MGs in high‐impact sports to prevent oral injuries. However, the evidence is not yet robust enough to mandate the use of performance‐enhancing MGs. Further research is needed in the form of larger, more rigorous studies to explore the specific mechanisms through which certain MGs may enhance the performance. Researchers should focus on standardizing methodologies, using more controlled crossover designs, and employing consistent performance metrics across different athletic populations.

## Author Contributions

Alice Cichelli and Rosita Delfina Valloreo substantially contributed to data collection, analysis, and drafting of the manuscript. Domenico Tripodi collaborated in data extraction and management, while Mirko Pesce supported the formal analysis. Simonetta D’Ercole contributed to the critical revision and editing of the manuscript. Maurizio Bertollo and Teresa Paolucci were responsible for the conceptualization of the study and supervised the entire research and writing process.

## Funding

This research received no external funding. Open access publishing facilitated by Universita degli Studi Gabriele dAnnunzio Chieti Pescara, as part of the Wiley ‐ CRUI‐CARE agreement.

## Disclosure

All authors have read and approved the final version of the manuscript.

## Ethics Statement

The authors have nothing to report.

## Conflicts of Interest

The authors declare no conflicts of interest.

## Data Availability

Data sharing is not applicable to this article as no datasets were generated or analyzed during the current study.
